# Editorial: Novel anti-cancer agents targeting tumour metastasis and stemness

**DOI:** 10.3389/fphar.2024.1504024

**Published:** 2024-10-30

**Authors:** Luping Pang, Bo Wang

**Affiliations:** ^1^ Department of Medical Genetics and Cell Biology, School of Basic Medical Sciences, Zhengzhou University, Zhengzhou, China; ^2^ School of Pharmaceutical Sciences, Zhengzhou University, Zhengzhou, China

**Keywords:** tumor, metastasis, stemness, novel anti-cancer strategy, cancer stem cells

Despite extensive efforts in drug development for cancer treatment, only a few successful therapeutic agents and strategies have emerged that specifically target patients with metastatic cancer, particularly those with distant metastasis. Cancer stem cells (CSCs), also known as cancer-initiating cells, represent a small population of cells within cancerous tissue that possess unlimited proliferative potential and the ability to drive tumor formation (Batlle and Clevers, 2017). CSCs are believed to be the primary cause of drug resistance, cancer relapse, and metastasis, largely due to their capacities for self-renewal and differentiation.

Importantly, CSCs can migrate to distant sites by promoting epithelial-to-mesenchymal transition (EMT), evading apoptosis, and escaping immune surveillance, all of which contribute to cancer metastasis and progression. As such, CSCs play a critical role in tumorigenesis, drug resistance, recurrence, and metastasis, highlighting the urgent need to develop targeted therapies to improve patient outcomes in oncology (Chu et al., 2024).

Currently, advanced secondary cancers are typically only detected after multiple metastases have already occurred. The challenges associated with detecting dormant cancer cells or small metastases further complicate cancer treatment. Additionally, drugs targeting cancer metastasis often exhibit high cytotoxicity, inconsistencies in patient outcomes, and contribute to the development of drug resistance. Therefore, there is an urgent need for the development of novel small molecules, biological drugs, and combination therapies that target key processes in cancer metastasis.

Several pathways, such as WNT/β-Catenin, Notch, PI3K/AKT, TGF-β, and PPAR, have been validated as key regulators of cancer metastasis. Effectively targeting these pathways offers promising opportunities for therapeutic interventions that could inhibit tumor growth and reduce cancer stemness (Yang et al., 2020). This research aims to identify innovative small molecules and effective therapeutic strategies to target tumor metastasis and CSC-related processes, with the ultimate goal of improving survival rates for cancer patients.

Metaplastic breast cancer (MpBC) is a Research Topic of morphologically diverse and exceedingly aggressive variant disease, which can be classified as high or low grade based on the pathological features. As one type of high-grade MpBC, squamous cell carcinoma (SCC) cases usually possess negative expression of estrogen receptor (ER), progesterone receptor (PR), and human epidermal growth factor receptor 2 (HER2), resembling triple-negative breast cancer (TNBC) but with a worse prognosis than conventional TNBC. Wang et al. reported an initial rare case of a patient diagnosed with HER2-positive breast SCC, who achieved favorable outcomes by utilizing HER2-targeted drugs in conjunction with chemotherapeutic drug paclitaxel. These findings suggest that HER2-targeted drugs can also exhibit positive effects on HER2-positive MpBC and SCC patients, thus providing a novel reference point for the subsequent treatment of uncommon pathological subtypes of breast cancer.

Recently, immune checkpoint inhibitors (ICIs) have revolutionized cancer treatment. However, a significant proportion of gastric cancer (GC) patients do not benefit from this therapeutic strategy. To elucidate the mechanisms underlying GC patients resistance to ICIs and identify biomarkers able to predict the response to ICIs at treatment initiation stage, Sun et al. collected GC tissues from 28 patients prior to the administration of anti-programmed death 1 (PD-1) immunotherapy and carried out protein quantification by high-resolution mass spectrometry (MS). Data analysis suggested that the low activity in the complement and coagulation cascades pathway and a high abundance of activated CD8^+^ T cells are positive signals corresponding to ICIs. They also identified 10 protein biomarkers with potential for predicting the response to PD-1 inhibitor immunotherapy in GC patients, which may provide the impetus for personalized precision immunotherapy.

For anti-cancer drug development, 2D cell culture is the most commonly used method to evaluate tumor metastasis capability, but with limitations in representing cancer hallmarks and phenotypes. Ding et al. reported an innovative approach which combines the advantages of 3D tumor spheroid culture with impedance-based biosensing technologies to establish a high-throughput 3D cell invasion assay for anti-metastasis drug screening through multicellular tumor spheroids. This method could be a promising tool for enhancing the quality of the drug development pipeline by providing a robust platform for predicting the efficacy and safety of anti-metastatic drugs before applying into preclinical or clinical trials.

To improve the therapeutic efficacy and selectivity of chemotherapeutic agents for cancer treatment, the nanodrug delivery and targeting systems are initiated and thrivingly developed in recent years. However, most nanocarriers exhibit drawbacks including an intricate preparation process, limited drug-loading capacity, untargeted drug release, and nanocarrier toxicities. Wang et al. took advantage of natural products with abundant scaffold diversity and structural complexity, and established two carrier-free berberine (BBR)-based nanoparticles (NPs) to increase their synergistic efficacy for tumor treatment. BBR interacts with glycyrrhetinic acid (GA) and artesunate (ART) to self-assemble BBR-GA and BBR-ART NPs, respectively, without any nanocarriers. These BBR-based NPs have been demonstrated to possess significant tumor targeting specificity and enhanced antitumor properties both *in vitro* and *in vivo*. These novel carrier-free self-assemblies based on natural products offer a strategy for synergistic drug delivery and thus shed lights on developing enhanced antitumor drugs.

The stemness of CSCs plays a key role in driving hepatocellular carcinoma (HC) tumorigenesis, apoptotic resistance, and metastasis while functional mitochondria are critical for the stemness maintenance. Cuproptosis is Cu-mediated non-apoptotic pathway by inactivating mitochondrial enzymes pyruvate dehydrogenase (PDH) and succinate dehydrogenase (SDH), leading to mitochondrial dysfunction. Thus, Abu-Serie et al. designed two types of Cu oxide nanoparticles (Cu_4_O_3_ “C(I + II)” NPs and Cu_2_O “C(I)” NPs) combined with an aldehyde dehydrogenase “ALDH” inhibitor diethyldithiocarbamate for anti-HC investigation. Being higher selective accumulation of the former NPs in tumor tissues, it exhibited higher cytotoxicity, mitochondrial membrane potential, and anti-migration impact than the later in the treated human HC cells.

Recently, long noncoding RNAs (lncRNAs) have been reported to be closely related to cancer metastasis. To explore whether lncRNA is also involved in the multi-organ metastasis in gastric cancer (GC), Zhang et al. conducted lncRNA-sequencing analysis of clinical lymph node metastatic GC tissues. They identified lncRNA CADM2-AS1 was aberrantly overexpressed in the metastatic GC tissues. Further mechanistic studies demonstrated overexpressed lncRNA CADM2-AS1 downregulated miR-5047 causing NOTCH4 upregulation to promote metastatic progression in GC. These results suggested that lncRNA CADM2-AS1 could be a potential effective target for metastatic GC prognosis in the clinic.

Non-coding RNAs (ncRNAs) are usually considered to be incapable of coding protein. However, the small peptide encoded by ncRNAs (SPENs) have been recently reported to play important roles in the invasive and migratory abilities of tumor cells, including the regulation of skeleton reorganization, intercellular adhesion, signaling and other processes. Therefore, SPENs have potential applications as therapeutic targets and biomarkers of malignant cancers. Liu et al. summaries the mechanisms of SPENs and their roles in tumor invasion and migration, with the goal of offering new targets for tumor diagnosis and treatment.

As mentioned earlier, CSCs are the leading cause of the anti-tumor treatment failure. These aggressive cancer cells can be preserved and sustained by adjacent cells constructing a specialized microenvironment, in which tumor-associated macrophages (TAMs) are key players. By improving the oxidative metabolism of CSCs and TAMs, cancer cells can extract more energy to survive in nutritionally defective environments. Among these metabolic pathways, mitochondria act as the crucial bioenergetic hub, drawing major hopes for drugs targeting mitochondria. Marrone et al. summarized the literatures on the metabolic adaptations of CSCs and their supporting macrophages, as well as highlighted the resistance and dormancy behaviors that give CSCs a selection advantage and quiescence capacity in particularly aggressive microenvironments and the critical role of TAMs in supporting these attitudes.

Despite extensive research on cervical cancer therapeutics, a bibliometric analysis specifically focused on immunotherapy for advanced, recurrent, or metastatic (A/R/M) cervical malignancies remains unmapped. Duan et al. conducted a systematic search using Web of Science Core Research Topic to identify articles related to A/R/M cervical cancer published between 2000 and 2022, providing a detailed landscape of immunotherapy research in A/R/M cervical cancer.

In conclusion, through multiple innovative approaches reported, this Research Topic advanced our understanding of the different mechanisms of cancer metastasis and CSCs in different cancer types and their potential drug targets and challenges. Continued research and novel strategies offer hope for investigating more effective therapeutics, eventually improving the life span and quality of cancer patients.
